# Accuracy of intraoral and extraoral digital data acquisition for dental restorations

**DOI:** 10.1590/1678-775720150266

**Published:** 2016

**Authors:** Heike Rudolph, Harald Salmen, Matthias Moldan, Katharina Kuhn, Viktor Sichwardt, Bernd Wöstmann, Ralph Gunnar Luthardt

**Affiliations:** 1 - Universität Ulm, Zentrum Zahn-, Mund-, und Kieferheilkunde, Klinik für Zahnärztliche Prothetik, Ulm, Germany.; 3 - Justus-Liebig Universität, Poliklinik für Zahnärztliche Prothetik, Giessen, Germany.

**Keywords:** Dental technology, Dental equipment, Systems analysis, Dental informatics

## Abstract

The computer-aided design (CAD) and computer-aided manufacturing (CAM) process chain for dental restorations starts with taking an impression of the clinical situation. For this purpose, either extraoral digitization of gypsum models or intraoral digitization can be used. Despite the increasing use of dental digitizing systems, there are only few studies on their accuracy.

**Objective:**

This study compared the accuracy of various intraoral and extraoral digitizing systems for dental CAD/CAM technology.

**Material and Methods:**

An experimental setup for three-dimensional analysis based on 2 prepared ceramic master dies and their corresponding virtual CAD-models was used to assess the accuracy of 10 extraoral and 4 intraoral optical non-contact dental digitizing systems. Depending on the clinical procedure, 10 optical measurements of either 10 duplicate gypsum dies (extraoral digitizing) or directly of the ceramic master dies (intraoral digitizing) were made and compared with the corresponding CAD-models.

**Results:**

The digitizing systems showed differences in accuracy. However, all topical systems were well within the benchmark of ±20 µm. These results apply to single tooth measurements.

**Conclusions:**

Study results are limited, since only single teeth were used for comparison. The different preparations represent various angles and steep and parallel opposing tooth surfaces (incisors). For most digitizing systems, the latter are generally the most difficult to capture. Using CAD/CAM technologies, the preparation angles should not be too steep to reduce digitizing errors. Older systems might be limited to a certain height or taper of the prepared tooth, whereas newer systems (extraoral as well as intraoral digitization) do not have these limitations.

## INTRODUCTION

Computer-aided design (CAD) and computer-aided manufacturing (CAM) technologies allow the use of a range of manufacturing methods and materials for dental restorations. Sophisticated high-strength materials, such as zirconium oxide, which can only be processed using CAD/CAM technologies, and generative (additive) technologies, such as rapid prototyping, require digital data as the key to apply any CAD/CAM process^29^.

The CAD/CAM process chain starts with taking an impression of the clinical situation, for example, by capturing a patient’s prepared tooth or teeth. Conventionally, high-precision condensation-curing or addition-curing impression materials are used to make dental impressions. Subsequently, dental stone is poured into these impressions. The resulting gypsum model can be digitized extraorally^23^. An ever more widely applied alternative is intraoral digitization^7,28^, which allows practitioners to dispense with conventional impressions and gypsum model fabrication. Both methods of data capture have advantages and disadvantages. Clinical parameters have the greatest impact on the quality of dental impressions^21^. These factors, which include moisture (saliva, blood), movement of patient and dentist, and restricted space in the oral cavity, can also impede intraoral digitizing. Because of space restrictions, intraoral digitizing devices have a smaller measuring area than extraoral digitizers. As a result, several digital data sets must be obtained and combined to capture more than two to three neighboring teeth when using intraoral digitizing devices. This process, termed matching, will always introduce a small systematic error to the data of any measuring system. However, conventional impressions of the full arch are also subject to greater deviation than those of single teeth. Furthermore, extraoral digitization always includes the errors introduced by impression and gypsum model-making in addition to the error resulting from digitization^27^. Regardless of the digitizing technology applied, digitization quality affects precision in the CAD/CAM process^2^. CAM precision also depends on the quality of the machining components^1,31^.

Every step in the CAD/CAM process chain can add to the resulting manufacturing error. The individual errors can cancel or mutually reinforce each other. Mixed effects are also possible^18^. With three-dimensional (3D) analyses, every step of the CAD/CAM process chain can be assessed, from intraoral digitizing or impression-making to the resulting dental restoration. Such analyses compare a real model with its corresponding virtual CAD model. Preferably, the real model is made of a directly digitizable material. Powder use can be avoided to reduce translucency or reflection, which will always increase error in the digitizing process^10,24^.

Data from every step in the CAD/CAM process chain, as well as from resulting digitized templates or restorations, can be aligned with the virtual CAD model and analyzed in 3D with suitable CAD software^3,9,12,19^. Very high standards for alignment must be set for these analyses. With alignment or registration, data sets are positioned in one common coordinate system with the least possible mean deviation^25^. This deviation is usually given in terms of the root mean square (RMS) error and should be documented for the assessment of systematic error in 3D analysis^5^.

Repeated measurement of reference models has been used to analyze the precision of digitizing systems^3,9,13,15^. However, geometrically defined reference models are of limited value for analyzing freeform surfaces. Because the surface of prepared teeth will render freeform surfaces after digitization and because tooth shape has a significant impact on achievable precision within a CAD/CAM process chain^6,15^, system precision should be analyzed with appropriately shaped reference models.

According to ISO 12836:2012^13^, definition 3.6, accuracy describes the closeness of agreement between the result of a measurement and the true value of the measurand. Accuracy is a qualitative concept. Its quantitative counterpart is trueness^13^. Precision is calculated as a standard deviation indicating the closeness of agreement among the results obtained when applying the experimental procedure several times under prescribed conditions^10,16^.

Despite the increasing use of dental digitizing systems, studies on their accuracy are lacking^6^. The current study compared the accuracy of various different intraoral and extraoral digitizing systems for dental CAD/CAM technology. The study’s hypotheses were that:

1. Optical non-contact digitizing systems for intraoral and extraoral data acquisition in CAD/CAM technology have differences in accuracy; and
2. The shape and type of tooth to be digitized affect the achievable accuracy, depending on the digitizing system used.

## MATERIAL AND METHODS

**Figure 1:**
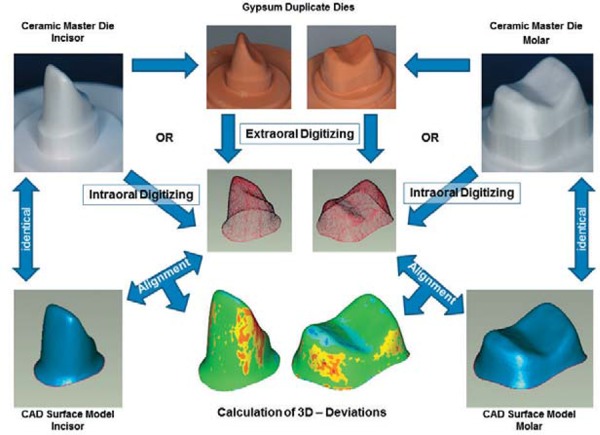
Experimental setup (reverse-engineering of the ceramic master dies and corresponding virtual computer-aided design (CAD) models excluded)

**Figure 2:**
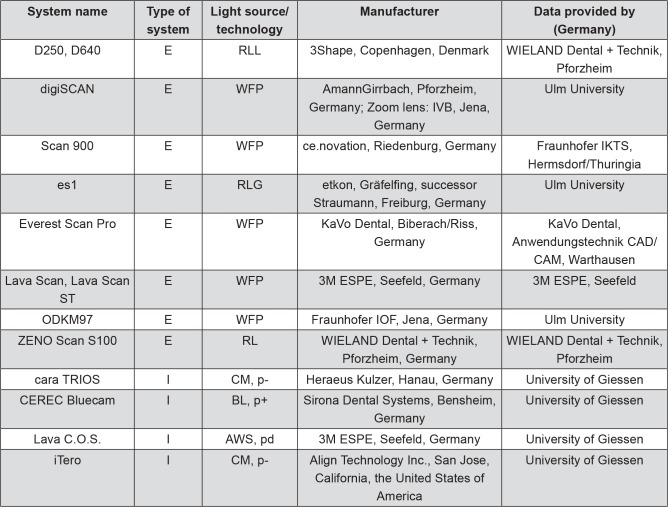
List of digitizing systems and system variations, type of system, applied light technology, manufacturer, and provider of digital data. I: intraoral; E: extraoral; RLL: red laser line; WFP: white-light fringe projection; RLG: red laser grid; R: red laser; CM: confocal microscopy; BL: blue laser; AWS: Active Wavefront Sampling; p+: with powder; p-: no powder; pd: powder dusting

An experimental setup for 3D analysis was used to assess and compare the accuracy of different dental digitizing systems, 10 for extraoral data acquisition (plus one system tested a second time using a zoom lens) and four for intraoral data acquisition (Figure 1, Figure 2). Analyses were based on two prepared ceramic master dies (molar and incisor) and their corresponding virtual CAD models.

### Reference digitizing system, analysis of precision

A high-precision optical white-light fringe projection digitizing system with a measurement uncertainty of ~8 µm (as given by the manufacturer) was chosen as reference scanner (ODKM97, Fraunhofer Institute for Applied Optics and Precision Engineering, IOF, Jena, Thuringia, Germany). In order to determine its precision, a preparatory study slightly differing in design was conducted. A steel canine (instead of the ceramic master dies) with a chamfer preparation was used for a series of 10 measurements of a gypsum duplicate die. All measurements were aligned to the CAD model corresponding to the steel canine. Afterwards, all measurements were three-dimensionally compared with one another using a CAD program (geomagic studio and qualify 10.0, 3D Systems, Darmstadt, North Rhine-Westphalia, Germany). Maximum and mean positive and negative deviations, standard deviation (SD), and the 95% Confidence Interval (CI) were calculated. The reliability of the repeated measurements was assessed by calculating Cronbach’s alpha.

### Reverse engineering of ceramic master dies and corresponding virtual CAD models

A molar and an incisor were prepared according to the rules for all-ceramic restorations (3M ESPE, Seefeld, Bavaria, Germany). The resulting gypsum dies were digitized with a high-precision optical white-light fringe projection digitizing system (ODKM97) with a measurement uncertainty of ~8 µm (according to the manufacturer). Using a CAD software program (ce.novation, ilmcad, Ilmenau, Thuringia, Germany), duplicate ceramic dies were constructed and CAD/CAM-made (Fraunhofer Institute for Ceramic Technologies and Systems, IKTS, Hermsdorf, Thuringia, Germany) by milling from partially sintered blocs (dispersion ceramic: zirconia 80%, alumina 20%). To eliminate surface reflection of the densely sintered dies, they were sandblasted with corundum abrasive (grain size 50 µm) at 2 bar.

The acquired (ODKM97) digital data sets (point clouds) from the ceramic dies were filtered to remove outliers and stray points. Corresponding virtual CAD models were generated from the point clouds by triangulation (“Points to Polygon” feature, Geomagic Studio). These reverse-engineered CAD models of the incisor and molar were used to analyze the measuring data from all digitizing systems evaluated (Figure 2).

### Extraoral digitizing

In accordance with common clinical and dental laboratory procedures, 10 gypsum dies (five molars and five incisors) were made from impressions for each of the systems/variations for extraoral digitizing. Each gypsum die was digitized once.

Impressions were made with the one-stage two-phase impression technique using addition-curing high-precision polyether (Impregum Penta H DuoSoft and Impregum Garant L DuoSoft; 3M ESPE, Seefeld, Bavaria, Germany) as well as individualized custom stock trays (KKD impression trays with retention, size 3; Kentzler-Kaschner Dental, Ellwangen/Jagst, Baden-Württemberg, Germany), which were covered with a thin layer of adhesive and dried for 10 minutes (Polyether-Adhesive; 3M ESPE, Seefeld, Bavaria, Germany). Because all impressions were made at room temperature rather than body temperature, the setting time recommended by the manufacturer was extended by 5 min.

Without using any surfactants, impressions were poured with vacuum-mixed class 4 stone (esthetic-rock 285, apricot; dentona DENTAL, Wipperfürth, North Rhine-Westphalia, Germany) following a standardized protocol^20,30^. Only flawless gypsum dies were used. Dies with bubbles or pearls were discarded and remade (impression and die). The bases of the dies were reduced by dry trimming only, to avoid water contamination of the gypsum.

All five molar and five incisor gypsum dies were digitized within 72 h of manufacture with the respective extraoral digitizing system (Figure 2). Following common clinical and dental laboratory procedure, system-specific standard provisions were used to improve data quality after digitizing. Data export format was either binary *.stl (stereolithography file format) or *.asc (ASCII: American Standard Code for Information Interchange), depending on the digitizing system.

### Intraoral digitizing

For all intraoral digitizing systems, the ceramic master dies were used directly for data acquisition, again according to clinical procedure, which does not include impression or gypsum model making. The incisor and the molar were digitized five times with each system. Whereas iTero and cara TRIOS (Figure 2) did not require the application of powder onto the ceramic master dies, CEREC Bluecam (VITA CEREC Powder; VITA Zahnfabrik, Bad Säckingen, Baden-Württemberg, Germany) did require powdering of the ceramic dies before digitizing. The Lava C.O.S. required neighboring and antagonistic teeth for the digitizing process. A slight dusting of powder (Powder for Chairside Oral Scanner; 3M ESPE|LAVA, Seefeld, Bavaria, Germany) was applied for pattern recognition and matching, according to the manufacturer’s instructions. Again, system-specific standard provisions were used to improve data quality after digitizing. Data export format was binary *.stl.

### Data handling and alignment

Each data set was oriented in its own measuring coordinate system. Before analysis, data sets were aligned to the respective virtual CAD model (molar or incisor) serving as reference (feature “best fit registration”, no point reduction chosen, Geomagic Qualify). The error in this alignment was recorded as RMS error prior to the analysis of the quality of fit.

The number of single points in each data set, representing the surface of the digitized tooth, depended in part on the size of the tooth. Incisor data sets consisted of 3,000 to 54,000 points, depending on the digitizing system (mean number of points per data set: ZENO Scan S100: 3,059; D640: 3,698; D250: 5,729; Everest Scan Pro: 9,156; es1: 13,841; CEREC Bluecam: 15,019; digiSCAN with zoom lens: 15,736; iTero: 15,888; cara TRIOS: 15,958; digiSCAN: 16,984; Lava C.O.S.: 17,586; Lava Scan: 29,525; Lava Scan ST: 30,216; ODKM97: 54,058). Molar data sets comprised 4,000 to 119,000 points (mean number of points per data set: ZENO Scan S100: 3,816; D640: 4,517; D250: 10,619; Everest Scan Pro: 12,229; es1: 18,649; CEREC Bluecam: 14,968; digiSCAN with zoom lens: 25,841; iTero: 21,179; cara TRIOS: 21,218; digiSCAN: 34,207; Lava C.O.S.: 24,310; Lava Scan: 40,550; Lava Scan ST: 39,854; ODKM97: 119,301). For better comparability, all data sets were sampled at a consistent inter-point distance of 50 µm, which reduced the number of points, especially in the larger data sets. After the procedure, incisor data sets consisted of 2,800 to 29,000 data points; molar data sets comprised 3,500 to 39,000 data points. Apart from this sampling, all data sets (point clouds or STL-surfaces) were used unchanged and exactly as exported by the respective digitizing system.

### Quantitative and qualitative analysis

For quantitative data analysis, the shortest distance between each point in a measurement point cloud and the reference was compared for each system/variation for each of the five incisor and five molar measurements. Maximum and mean positive and negative deviations from the virtual CAD master model were calculated (Geomagic Qualify). Color-coded graphs (Geomagic Qualify) were used to show how the calculated differences were spread over the complete die surface, thus revealing strengths and weaknesses of each digitizing system.

### Statistical analysis

Data were analyzed descriptively with one-way analysis of variance (ANOVA; IBM SPSS 21.0; IBM Corporation, Armonk, NY, USA) and the Student–Newman–Keuls *post-hoc* procedure, at a level of significance of α=0.05. Factors for the multivariate analysis of variance (MANOVA) were “tooth” (incisor versus molar) and “system” (the 15 digitizing systems/variations).

## RESULTS

### Reference digitizing system, analysis of precision

Comparing each of the 10 measurements obtained by ODKM97 with one another resulted in a mean deviation of +6.0 µm (SD 1.2) and -5.3 µm (SD 1.4). The maximum positive deviation was +8.8 µm and the maximum negative deviation was -10 µm. The confidence interval (CI) for the mean positive deviation was 5% CI 4.2 to 95% CI 8.3 µm and the CI for the mean negative deviation was 5% CI -8.2 to 95% CI -3.4 µm.

The value for Cronbach’s alpha was 0.999*.*

### Precision of data alignment

For most digitizing systems, an RMS error of 10±3 µm was achieved for data alignment with the reference CAD model (maximum 21 µm, minimum 5 µm). Only D250 (RMS 18±7 µm; maximum 31 µm; minimum 10 µm), digiSCAN with zoom lens (RMS 31±14 µm; maximum 54 µm; minimum 10 µm), and Scan 900 (RMS 37±12 µm; maximum 51 µm; minimum 24 µm) had less exact alignment values.

### Quantitative analysis

**Figure 3:**
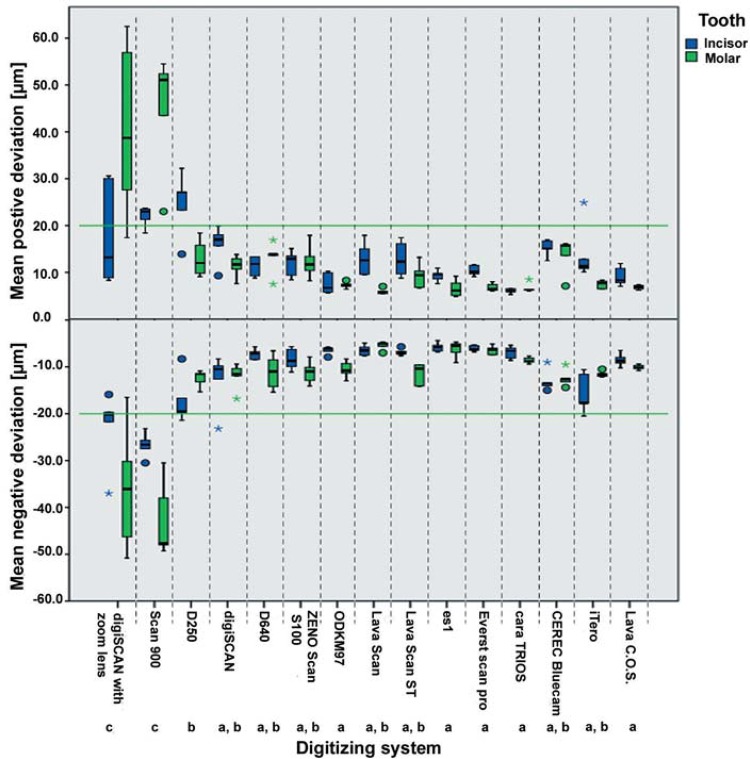
Boxplot showing mean positive and negative deviations according to digitizing system and tooth. Outlier values are more than 1.5 times the box width away from the boxes’ outer boundaries and indicated by circles. Extremes are more than 3 times the box width away from the boxes’ outer boundaries and indicated by asterisks. Systems indicated with the same letters (a, b, c) show no significant statistical difference

Quantitative data analysis rendered the positive and negative deviations for each of the 10 measurements for each of the 15 digitizing systems/variations. The effect of different tooth shape (incisor versus molar) was also determined (Figure 3).

### Qualitative analysis

**Figure 4:**
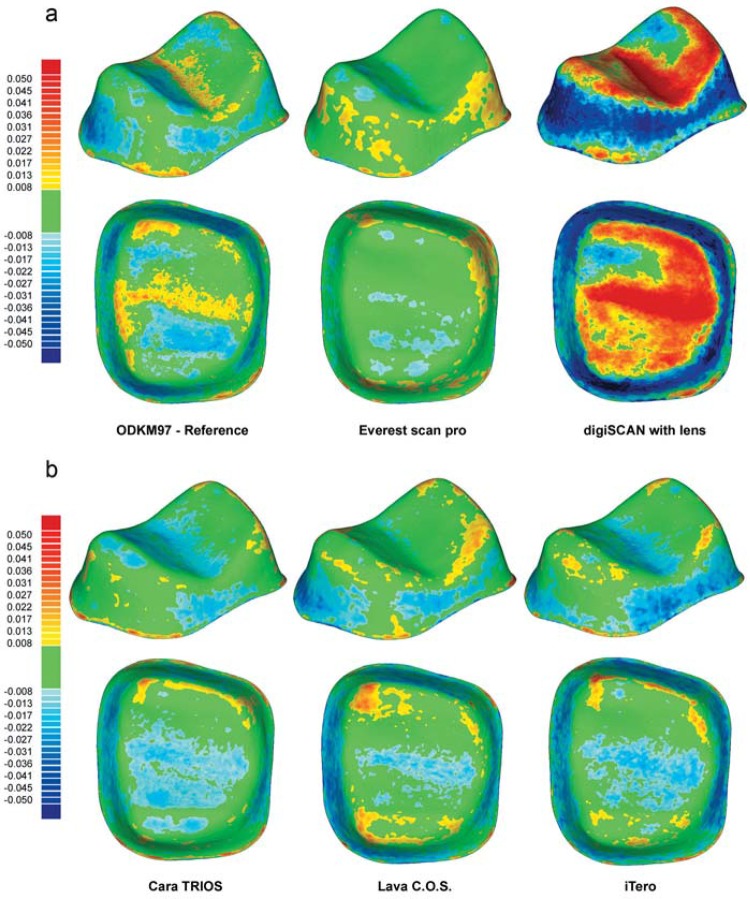
Qualitative, color-coded graphical analysis of extraoral (a) and intraoral (b) digitizing systems capturing the ceramic master molar

Among extraoral digitizing systems, ODKM97, Lava Scan, Lava Scan ST, es1, and Everest Scan Pro had the most precise reproduction of the ceramic master molar (Figure 4a, left and middle). Rather than improving the digitization results, the addition of the zoom lens to the digiSCAN system resulted in decidedly poorer digitizing quality (Figure 4a, right). The zoom lens affected the molar measurements more than the incisor measurements. The insignificant differences between extraoral (Figure 4a, left and middle) and intraoral digitizing systems (Figure 4b) in measuring the ceramic master molar can be assessed at a glance with the color-coded graphs.

**Figure 5:**
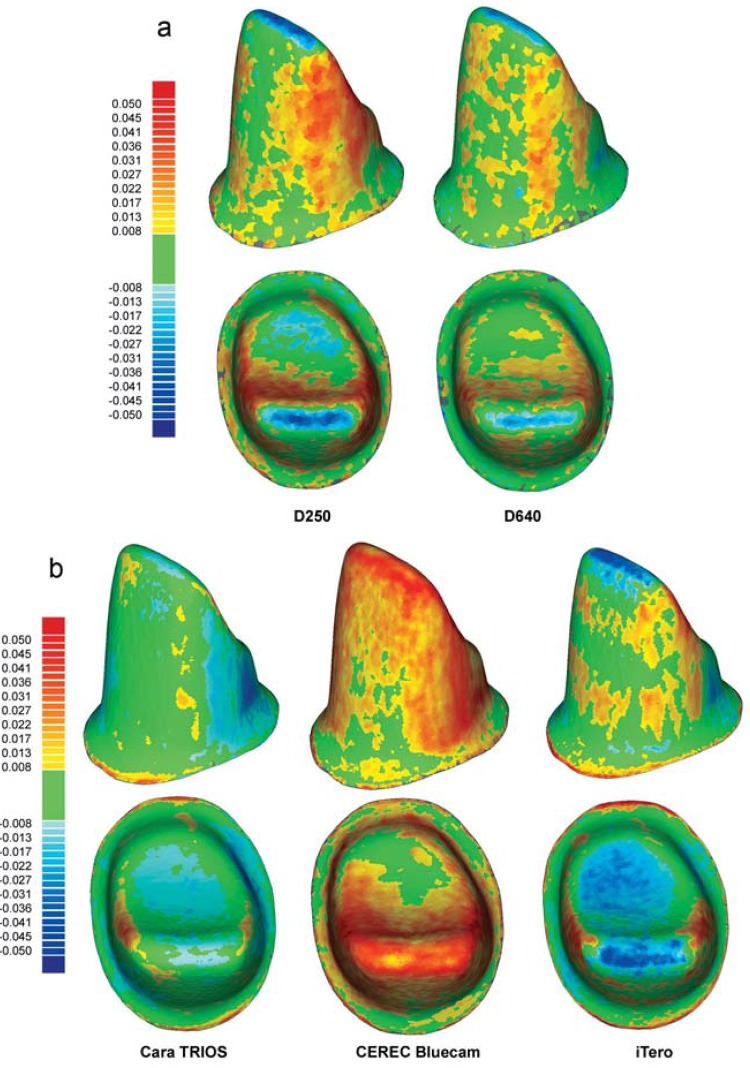
Qualitative, color-coded graphical analysis of extraoral (a) and intraoral (b) digitizing systems capturing the ceramic master incisor

The ceramic master incisor proved to be more challenging than the molar for most of the digitizing systems examined. The only exceptions were the D640, ZENO Scan S100, ODKM97, and the intraoral system cara TRIOS (Figure 5b, left). Most systems had higher deviations in the incisor data sets than in the molar measurements (Figures 5a and b, middle and right). The powder, which had to be applied before using the CEREC Bluecam system, clearly showed in the resulting higher positive deviations (Figure 5b, middle).

While two generations of the same digitizing system (D250 and D640) did not render different results for the molar measurements, there was a clear improvement with use of the newer model for the incisor measurements (Figure 5a).

### Statistical analysis

One-way ANOVA at a level of significance of α=0.05, followed by the Student–Newman–Keuls *post-hoc* procedure using the mean positive and negative deviation for analyses, showed that the highest digitization quality regarding positive deviations was achieved by the extraoral digitizing systems ODKM97 (7.5±1.6 µm), es1 (7.9±2.0 µm), and Everest Scan Pro (8.6±2.1 µm). Comparable, not significantly differing quality (p=0.327) was found for the intraoral digitizing systems Lava C.O.S. (8.0±1.9 µm) and cara TRIOS (6.4±0.8 µm). The extraoral systems Lava Scan (9.4±4.4 µm), Lava Scan ST (11.1±3.6 µm), ZENO Scan S100 (12.1±3.1 µm), D640 (12.2±2.9 µm), and digiSCAN (13.7±4.0 µm) also had high and not significantly differing (p=0.327) digitization quality, as did the intraoral systems iTero (10.7±5.4 µm) and CEREC Bluecam (14.5±2.9 µm). Only D250 (because of the incisor measurements, 18.9±8.1 µm, p<0.001), digiSCAN with zoom lens (due to the molar measurements, 29.4±18.9 µm, p<0.001), and Scan 900 (33.4±14.9 µm, p<0.001) rendered data sets of lower quality compared with the abovementioned digitizing systems.

Results for the mean negative deviations were similar, with best results (-6.0±1.1 to -8.5±2.5 µm) for the extraoral systems Lava Scan, es1, Everest Scan Pro, and ODKM97 as well as the intraoral system cara TRIOS (-7.7±1.3 µm). Only the mean negative deviations of D250 (-14.8±4.4 µm) and iTero (-13.6±3.6 µm) were found to be significantly larger (p<0.001) compared with the previously mentioned systems and Lava Scan ST, D640, ZENO Scan S100, and digiSCAN (extraoral) as well as Lava C.O.S. and CEREC Bluecam (intraoral).

Again, a lower quality in comparison with all other systems (p<0.001) was shown by digiSCAN with zoom lens and Scan 900 (-29.4±12.6 µm and -34.7±10.2 µm, respectively).

MANOVA revealed a significant influence of the combined factors “system” and “tooth” (p<0.001). The effect size, measured by calculating the partial eta-squared (η^2^_p_) value, was 0.480 (p<0.001). The digitizing system itself proved to have the greatest influence on the digitization results (η^2^_p_=0.713, p<0.001), while the factor “tooth” itself had little effect (η^2^_p_=0.001, p=0.749).

## DISCUSSION

This experimental setup allowed a broad comparison of different technological concepts for extraoral and intraoral digitizing systems. The method of analysis was developed from a study design originally used to evaluate 3D changes in gypsum dies over time^20^.

One major advantage of dispersion ceramic dies is their non-reflecting surface, compared with the metal dies used in prior studies^15,20^.

The aspects of the respective clinical procedure were considered implying gypsum duplicate dies for the extraoral systems and digitizing the ceramic master die directly with the intraoral systems. The small but existing error of impression and gypsum die making of single teeth^30^ raises the systematic error in relation to intraoral digitizing. However, eliminating this bias from the experimental set-up would have meant to add an error source to the intraoral procedure that is not part of the clinical procedure. At the same time, one of the advantages of intraoral digitizing would have been precluded.

Dispersion ceramic dies can be directly digitized without the use of powder (unless required by the digitizing technology itself), which reduces the systematic error of the experimental setup^8^. It cannot be ruled out that the ceramic material influenced the results either positively or negatively compared with dentine since especially the presence of water affects the digitizing quality greatly^17^. Looking at the quite similar results for single teeth (premolars) in a clinical trial using the CEREC 3D intraoral digitizing system^27^ (a system that was not part of the study at hand) suggests that the material influence in the *in-vitro* set-up presented here is probably of minor importance.

The only system requiring complete powdering in this study was the CEREC Bluecam. The powder thickness of approximately 20 to 40 µm has to be considered^24^ when comparing these values with the other intraoral systems. In the clinical procedure, knowing the approximate powder layer thickness allows adjustments for these deviations in the subsequent steps of the process chain. Applying the powder as evenly as possible is mandatory for high digitization precision. Powdering is prone to application errors^8^.

The data sets obtained with the digitizing system that needed slight powder dusting for pattern recognition (Lava C.O.S.) did not show an additional layer or thickness in this *in vitro* investigation.

In addition to the use of non-reflecting dies, reverse-engineering the virtual CAD model and corresponding real master die omits manufacturing error^22^ and thus improves the method of analysis.

A very high precision and consequent reliability (standard deviation of the mean positive deviation/mean negative deviation of the repeated measurements) of SD=0.0012/0.0014 mm and a very good reliability (Cronbach’s alpha=0.999) of the repeated measurements was found for the reference digitizer ODKM97. Reference digitizers should be of high precision for the accuracy analysis of dental digitizing systems^15^.

The number of points in a single tooth data set is not necessarily interlinked with the achievable precision. After digitizing, the original point density may be reduced because of data size reduction (e.g., in order to save storage space or to enable fast data transfer). Because of the constantly evolving development in storage capacity and high speed connections, this aspect has become less important today. However, systems based on the same basic technology (D250, D640, ZENO Scan S100), which are rendering low density data (around 3,500 points *per* data set depending on the system and tooth), showed comparable results to intraoral and extraoral systems delivering data sets with an order of magnitude higher number of points (digiSCAN, Lava Scan, Lava Scan ST, CEREC Bluecam, iTero). Reporting positive and negative mean deviations separately instead of absolute values allows for clearly differentiating between enlargement and reduction. The confidence intervals found for positive and negative deviations for a digitizing system may quite differ in size. However, looking at the absolute values in this study did not change the results. Statistically significantly (p<0.0001) different absolute deviations were shown by D250 (absolute mean 16.8 µm), digiSCAN with zoom lens (absolute mean 29.4 µm) and Scan 900 (absolute mean 34.1 µm).

Because group sample sizes were equal in this *in vitro* study, partial eta-square was used to estimate the effect size. However, the interpretation of these values according to a rule of thumb (small 0.2, middle 0.5, and large 0.8) should not be over-interpreted; the values are only intended to give a sense of the size of the effect of interest.

Based on the statistical results, the first hypothesis (that optical non-contact digitizing systems for intraoral and extraoral data acquisition in CAD/CAM technology have differences in accuracy) can be accepted. Topical systems for both extraoral and intraoral digitization are well below the benchmark of ±20 µm derived from *in vitro* analyses of conventional impression and die-making precision^21,30^. In terms of the digitization technology, high quality data could be obtained with white-light fringe projection, red or blue lasers, projected laser lines or grids, confocal microscopy, and Active Wavefront Sampling. These results are supported by a recent review on intraoral digitizing systems^32^.

As found in previous studies^14^, the shape of a prepared tooth, which is represented by the factor “tooth” in the present study, was found to affect the achievable accuracy, depending on the digitizing system used. Thus, the second hypothesis was confirmed.

The factor “tooth” should not be taken literally. Rather, the incisor or molar preparations represent different angles (obtuse or acute) and shadowed regions, which cannot be digitized accurately or steep and parallel opposing tooth surfaces. For most digitizing systems, steep and parallel incisor surfaces are generally the most difficult areas to capture. This effect can be seen in Figure 3, which shows that most of the digitizing systems have clearly greater accuracy when digitizing molars. Depending on the individual preparation, the problems with steep preparation angles can also occur with molars^15^ in convergence angles of 0°^6^. These problems were less pronounced for the high-accuracy digitizer (±10 µm) analyzed in the study of Chan, et al.^6^ (2011) than for lower-accuracy systems (20–40 µm). The influence of the convergence angle becomes more pronounced further along the CAD/CAM process chain^4^.

The successful compensation for such systematic error introduced during incisor digitization can be seen when comparing the more recent 3Shape digitizer model (D640) with the older model (D250). Weaknesses such as difficulties with steep and parallel surfaces can be detected with the experimental setup presented here.

The current results are limited because only single teeth were used^1^. Conclusions concerning long-span digitization^11,26^ and suitable digitizing strategies^10^ cannot be drawn. However, the use of individual teeth is an advantage when broadly comparing many different digitizing systems and technologies.

Results of previous digitizing system analyses have been reported in many different variations: positive and negative deviations^12^, absolute values^26^, point to point measurements, different surface areas, deviation percentages, mean values for accuracy^9^, and combinations thereof, which make it difficult to compare results across studies. The application of ISO 12836:2012^13^ could lead to better comparability of future results.

## CONCLUSIONS

Study results are limited since only single teeth were used for comparison. Optical non-contact digitizing systems for intraoral and extraoral data acquisition in CAD/CAM technology showed differences in accuracy, but all topical systems were well below a benchmark of ±20 µm.

The incisor or molar preparations used represent different angles as well as steep and parallel opposing tooth surfaces in the incisor preparation. For most digitizing systems, the latter are generally the most difficult areas to capture. When using CAD/CAM technologies, the preparation angles should not be too steep and sharp edges should be rounded off to reduce digitizing errors. Older systems might be limited to a certain height or taper of prepared tooth. More recent systems for extraoral, as well as for intraoral digitization, do not have these limitations.
